# No significant change in tibial torsion despite hinge fracture after high tibial osteotomy using patient‐specific instruments

**DOI:** 10.1002/jeo2.70960

**Published:** 2026-10-26

**Authors:** Luca Maddaloni, Lukas Jud, Lukas Fischer, Lazaros Vlachopoulos, Sandro F. Fucentese

**Affiliations:** ^1^ Department of Orthopedics Balgrist University Hospital, University of Zurich Zurich Switzerland

**Keywords:** hinge fracture, open wedge osteotomy, patient specific instruments, tibial torsion

## Abstract

**Purpose:**

Medial open wedge high tibial osteotomy (MOW‐HTO) is an established treatment option for medial knee osteoarthritis or overload. However, using the conventional technique, unintended postoperative changes of the tibial torsion angle (TTA) were observed, potentially leading to secondary patellofemoral or gait disorders. This study aimed to investigate postoperative TTA changes after MOW‐HTO performed with patient‐specific instruments (PSI), with particular attention to the influence of hinge fracture.

**Methods:**

All patients who underwent MOW‐HTO from October 2014 to December 2024 were included retrospectively. Pre‐ and postoperative radiographs and computed tomography (CT) were used to screen for HF and changes in TTA, posterior tibial slope (PTS), hip‐knee‐ankle angle (HKA) and opening wedge distance (OWD) were measured.

**Results:**

One hundred and eighteen knees were included, in which 52 HF (44.1%) were detected and classified according to Takeuchi (type I: *n* = 40, type II = 11, type III = 1). Overall, TTA showed no significant change from pre‐ to postoperative (25.1° ± 8.5°, 25.0° ± 8.6°, *p* = 0.727). HF group showed no significant change in TTA (24.3° ± 8.6°, 24.2° ± 9.0°, *p* = 0.803). Furthermore, HF group has significantly larger OWD values than patients in the no HF group (no HF group: 7.4 mm ± 2.1 mm, HF group: 9.2 mm ± 2.9 mm, *p* < 0.001). PTS changed significantly in the HF group (no HF group: 0.3° ± 1.9°, HF group: 2.0° ± 2.6°, *p* < 0.001).

**Conclusion:**

No significant change in TTA was observed in MOW‐HTO performed with a PSI system consisting of an osteotomy guide followed by a reduction guide. TTA also remained stable in cases with a hinge fracture.

**Level of Evidence:**

Level III.

## Graphical abstract

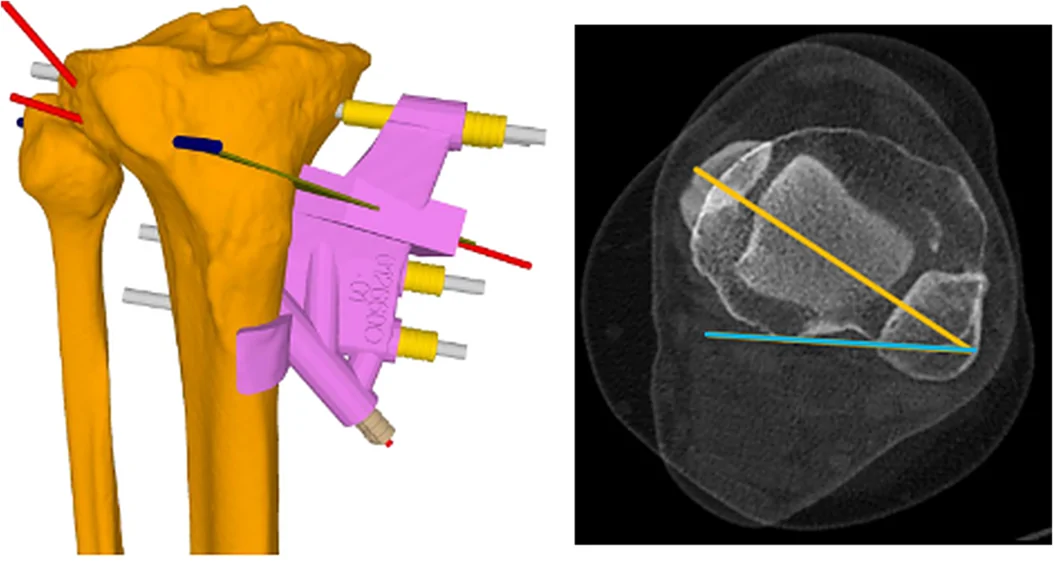

**Abbreviations**
CI: confidence interval
CT: computed tomography
HF: hinge fracture
HKA: hip‐knee‐ankle angle
ICC: intraclass correlation coefficient
LLR: long leg radiographs
MOW‐HTO: medial open wedge high tibial osteotomy
OWD: opening wedge distance
PSI: patient specific instruments
PTS: posterior tibial slope
TTA: tibial torsion angle

## INTRODUCTION

The main indication for a medial open wedge high tibial osteotomy (MOW‐HTO) is degeneration or overload of the medial compartment in the varus knee of symptomatic young patients [3, 25, 26, 28]. The intention of MOW‐HTO is joint preservation or at least to delay the need for knee arthroplasty, and long‐term outcomes show a joint preservation rate of two‐thirds over 12 years with high patient satisfaction [1]. MOW‐HTO can be performed with different techniques, one of them using patient‐specific instruments (PSI) [7, 8, 13, 14]. PSIs have been shown to improve surgical accuracy and precision compared to conventional techniques [11, 13, 28]. After MOW‐HTO unintended changes in the tibial torsion angle (TTA) and the posterior tibial slope (PTS) have been reported despite the use of PSI [17, 29]. Such rotational changes are clinically relevant, as unintended tibial rotation may alter lower limb kinematics and patellofemoral tracking, potentially resulting in gait abnormalities and anterior knee pain [1, 3, 34]. Lateral cortical instability, as observed in HF, has been identified as one of the main causes of unintended TTA changes [17].

The aim of this study was to evaluate postoperative TTA changes after MOW‐HTO performed with PSI system, with and without hinge fracture. It was hypothesised that postoperative changes in TTA are small and not substantially different between cases with and without HF in this PSI cohort. Secondarily, it was assumed that a MOW‐HTO technique with a descending osteotomy behind the tibial tuberosity with additional screw fixation leads to a smaller deviation of TTA than the common MOW‐HTO technique with an ascending osteotomy behind the tibial tuberosity [2].

## MATERIALS AND METHODS

### Patient selection

This retrospective single‐arm observational study was approved by the local ethical committee (Zurich Cantonal Ethics Commission, BASEC‐Nr. 2023‐00389). All patients who underwent isolated medial MOW‐HTO from October 2014 to December 2024 at the study institution were retrospectively included. An additional inclusion criterion was the availability of a pre‐ and postoperative full radiographic dataset comprising conventional knee radiographs, long leg radiographs (LLR) and a computed tomography (CT) scan. The CT scan was performed preoperatively for surgical planning and 4.5 months after surgery, which is a standard procedure at the study institution, to assess the state of consolidation of the osteotomy. Exclusion criteria were concomitant surgical procedures (e.g., PTS correction), previous surgery or osseous injury of the knee joint, and incomplete radiographic data.

Preoperative 3D planning was performed based on CT data as previously described. The correction was planned individually, considering the underlying indication. In most cases, a neutral to slightly valgus mechanical axis was targeted. The final extent of correction was determined by the treating surgeon based on weight‐bearing long‐leg radiographs and the patient's clinical presentation. The osteotomy was referenced to a lateral tibial hinge point located 15 mm below the tibial plateau and 10 mm medial to the lateral cortex [8, 14]. Intraoperatively, the patient‐specific osteotomy guide was placed and secured with four 4.0 mm Schanz pins. The osteotomy was then performed according to the predefined plane, orientation, and depth. The reduction guide was applied over the four previously placed Schanz pins, achieving the planned opening wedge distance (OWD). Finally, the osteotomy was fixed using the TomoFix Medial High Tibial Plate (DePuy Synthes) [8] (Figure 1). Regarding the osteotomy behind the tibial tuberosity, two techniques were used: the conventional MOW‐HTO technique with an ascending osteotomy behind the tibial tuberosity, and a descending osteotomy behind the tibial tuberosity with additional screw fixation (Figure 2). The latter technique was performed in cases requiring large corrections, in patients with preoperative patella baja, or when patellofemoral symptoms were present.

**Figure 1:**
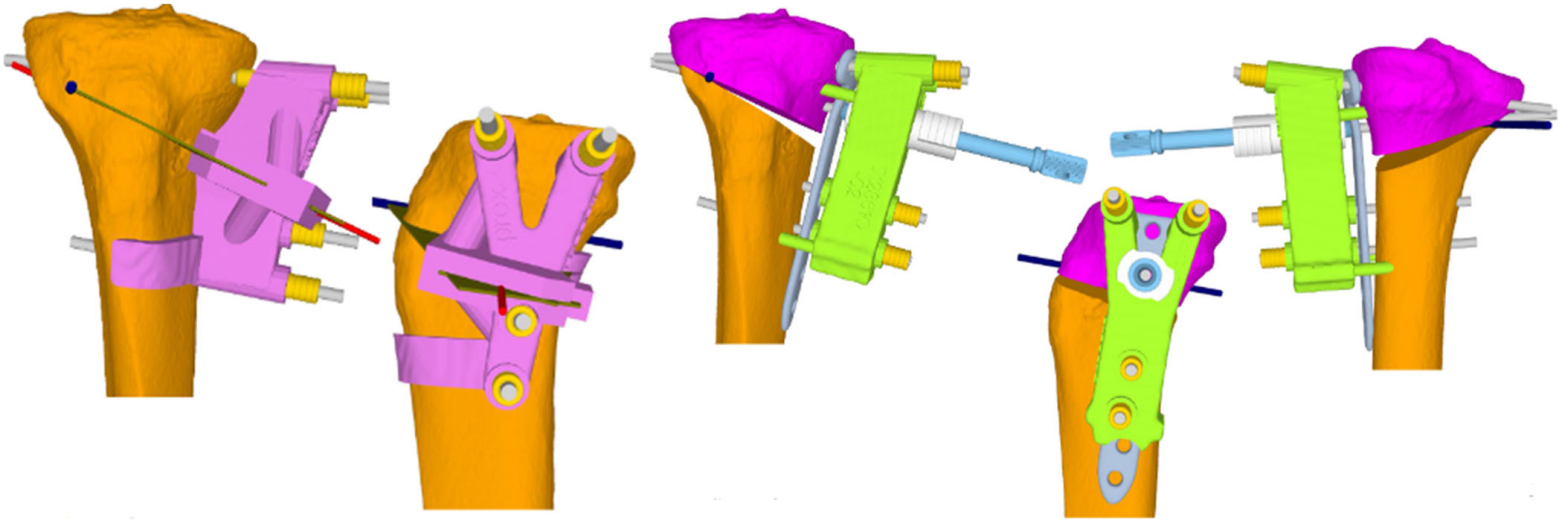
Patient‐specific instruments (PSI) system used in this study. Left: The osteotomy‐guide (purple) is fixed using four Schanz pins (grey) and reduction sleeves (yellow). A K‐wire (red) is used to control the correct guide positioning under fluoroscopy. Right: The TomoFix plate (grey) and the reduction guide (green) are placed over the previously set Schanz pins using reduction sleeves (yellow) for a rigid construct stability.

**Figure 2:**
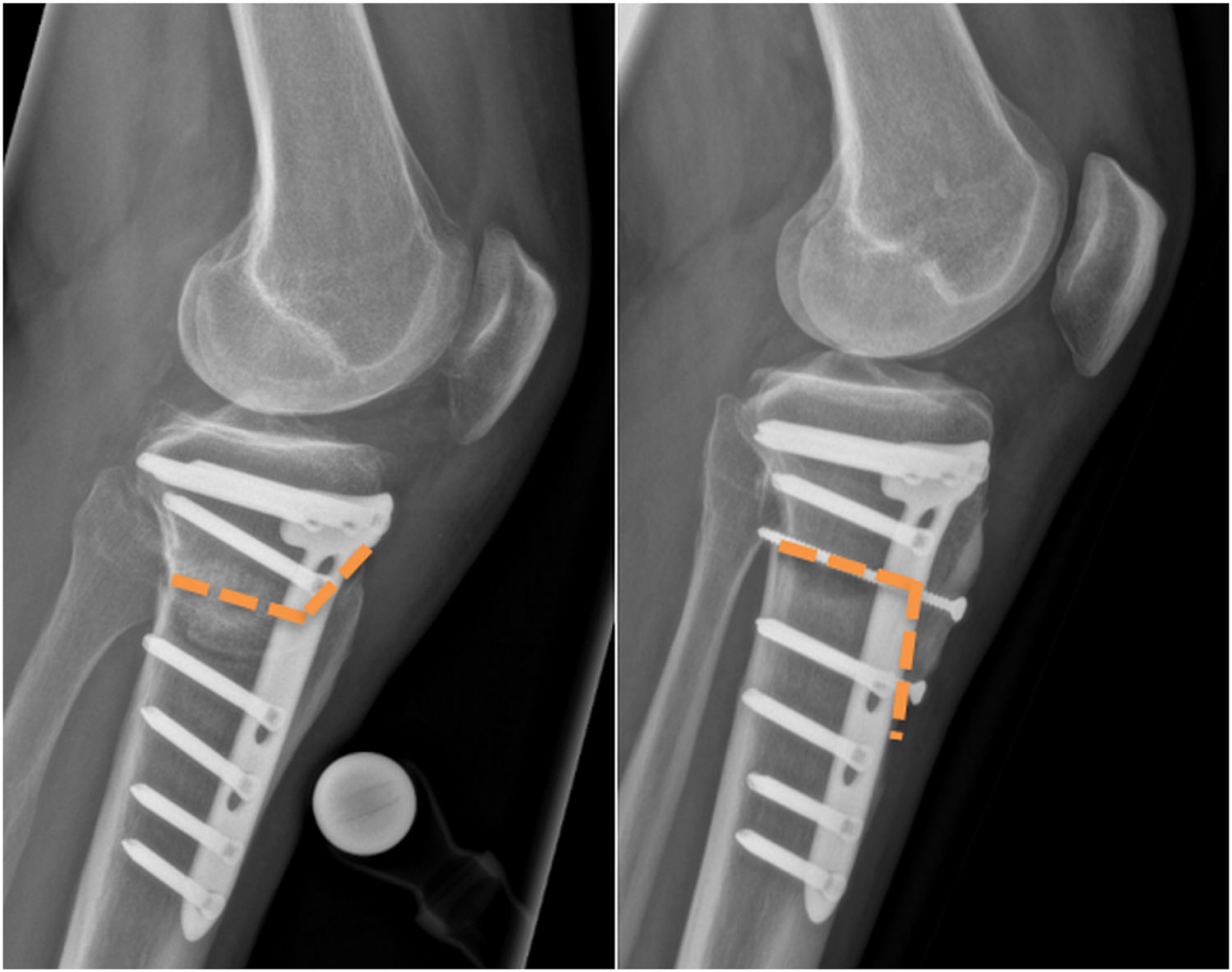
Representative lateral radiographs of two patients following medial open wedge high tibial osteotomy. Orange dashed lines indicate the osteotomy trajectories. Ascending osteotomy behind the tibial tuberosity (left), and a descending osteotomy behind the tibial tuberosity (right).

### Radiological assessment

The following radiological parameters were evaluated in pre‐ and postoperative images: TTA was measured using axial CT data as the angle between the tangent to the posterior tibial plateau and the bimalleolar axis according to Goutallier et al. [9] (Figure 3). PTS was measured using lateral knee radiographs as the angle between the proximal midline axis of the tibia and the tangent to the medial tibial plateau [6]. Hip‐knee‐ankle angle (HKA) was measured in LLR according to Jud et al. [16]. OWD was measured in coronal CT data as the largest distance of the osteotomy gap. HF were classified according to Takeuchi using postoperative radiographs and CT [35] (Figure 4). All parameters were analysed by two independent readers (L.M. and L.F.) for inter‐rater reliability, for the statistical analysis the mean between the two readers was used.

**Figure 3:**
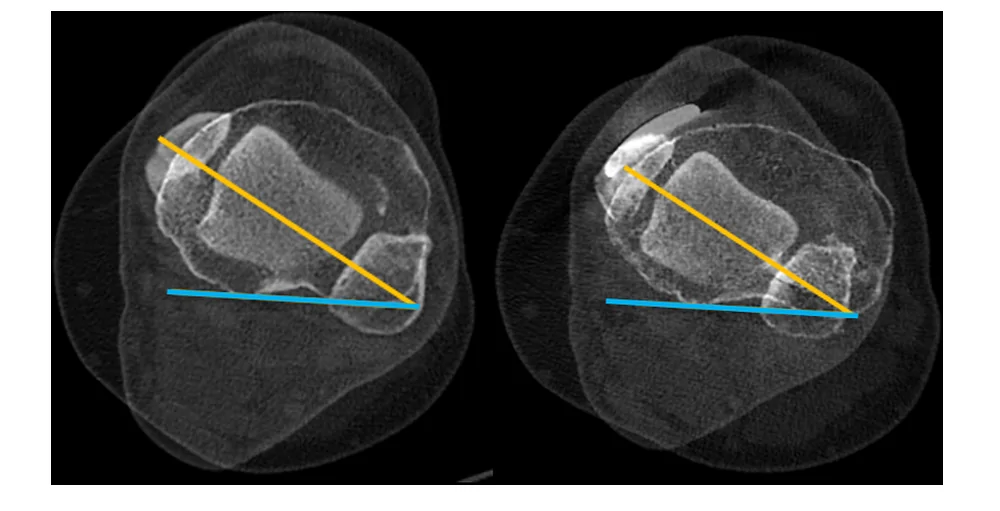
Measurements of the tibial torsion angle in pre‐ and postoperative computed tomography as the angle between the tangent to the posterior tibial plateau (blue) and the bimalleolar axis (yellow) [7]. Left: preoperative, 22.6°. Right: postoperative, 23.1°.

**Figure 4:**
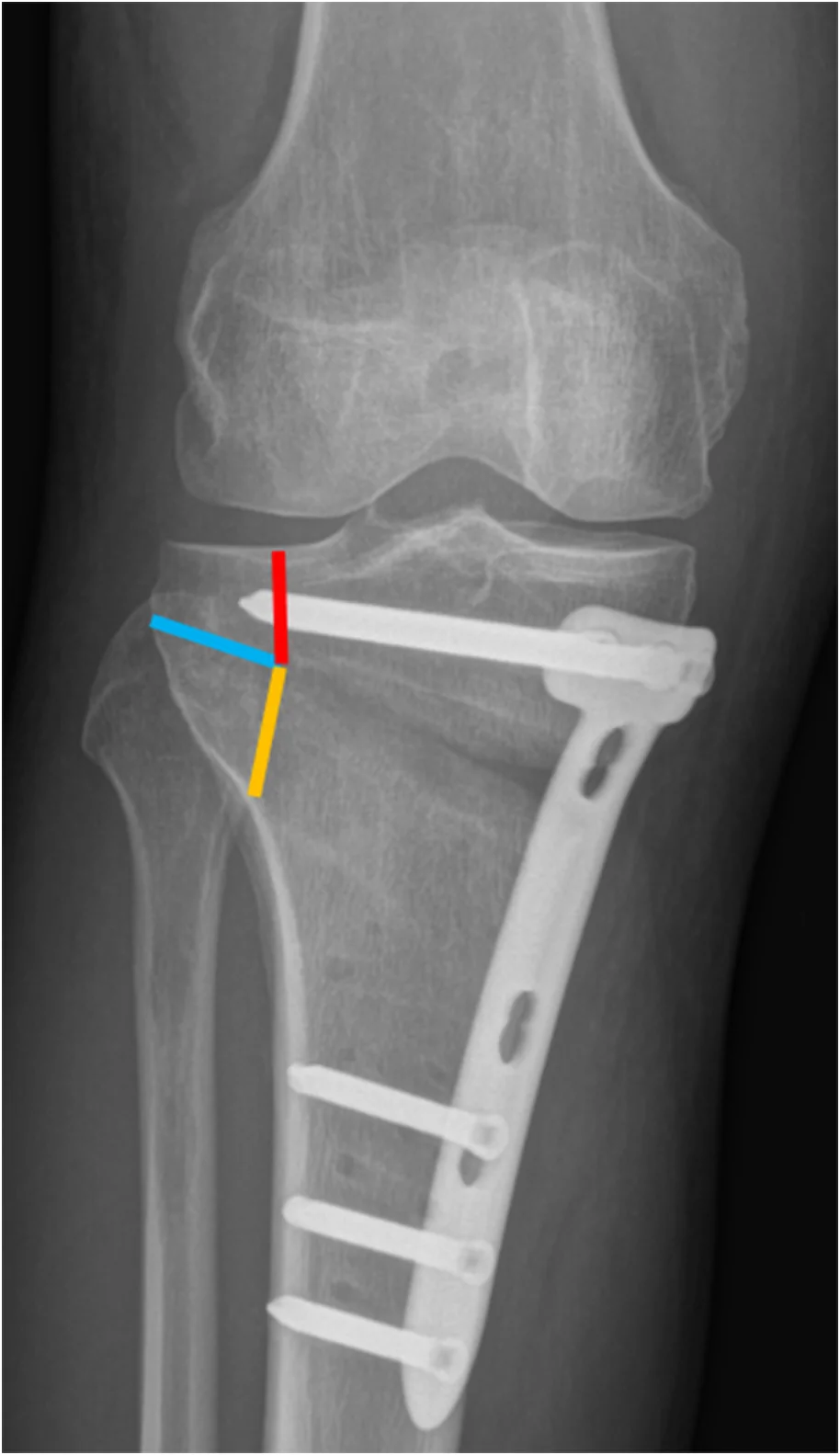
Classification of hinge fractures according to Takeuchi et al. [35], shown on an anteroposterior radiograph after medial open wedge high tibial osteotomy (MOW‐HTO). Type I (blue): fracture line extending to the proximal tibiofibular joint. Type II (yellow): fracture line extending distal to the proximal tibiofibular joint. Type III (red): fracture line extending into the lateral tibial plateau.

### Statistical analysis

Demographic and clinical characteristics of patients were summarised using descriptive statistics. Continuous variables are reported as mean and standard deviation (SD). The difference between preoperative and postoperative TTA, HKA and PTS was calculated for each case. The data were then primarily grouped into no HF and HF with further exploratory subgroup stratification according to Takeuchi. Lastly, ascending and descending osteotomies behind the tibial tuberosity were compared. Normality of distribution was tested using the Shapiro–Wilk test. Accordingly, the paired *t*‐test or Wilcoxon‐test was performed within the groups. To compare the groups, the unpaired *t*‐test or Mann–Whitney‐*U*‐test was applied. Inter‐rater reliability was calculated with the intraclass correlation coefficient (ICC). Inter‐rater agreement for the ordinal Takeuchi classification (grades 0–3) was assessed using weighted Cohen's kappa [5]. ICCs were interpreted according to Landis and Koch [21]. All statistical analyses were performed in SPSS for Windows (Version 31.0, SPSS Inc.).

## RESULTS

One hundred and eighteen knees (58 left, 60 right) in 110 patients (25 females, 85 males) were included. The mean age was 44.4 ± 9.7 years, and mean body mass index (BMI) was 28.6 ± 4.8 kg/m^2^. In 52 cases (44.1%), a HF was detected. This included 40 type I (34%), 11 type II (9%) and 1 type III (1%) HF. In 56 cases, an ascending and in 62 cases a descending osteotomy behind the tibial tuberosity was used.

TTA showed no significant changes from pre‐ to postoperative for all groups, the findings are summarised in Table 1. Mean changes of TTA from pre‐ to postoperative revealed no significant changes and are summarised in Table 2. PTS showed no significant change from pre‐ to postoperative in the no HF group (8.0° ± 4.0° to 8.2° ± 3.6°, *p* = 0.616), whereas a significant change was observed in the HF group (7.7° ± 3.7°, 9.6° ± 4.2°, *p* < 0.001). The mean change in PTS differed significantly between the groups, with a greater change in the HF group (2.0° ± 2.6°) than in the no HF group (0.3° ± 1.9°, *p* < 0.001). HKA changed significantly from pre‐ to post‐operative in both groups (no HF group: 173.0° ± 3.3° to 181.2° ± 2.2°, *p* < 0.001, HF group: 171.6° ± 3.9° to 180.1° ± 2.8°, *p* < 0.001).

**Table 1:** Pre‐ and post‐operative tibial torsion angle (TTA) (mean value ± standard deviation).

	TTA preop (°)	TTA postop (°)	*p*‐Value
Overall (*n* = 118)	25.1 ± 8.5	25.0 ± 8.6	0.727
No HF group (*n* = 66)	25.9 ± 8.5	25.8 ± 8.4	0.809
HF group (*n* = 52)	24.3 ± 8.6	24.2 ± 9.0	0.803
Takeuchi I (*n* = 40)	23.4 ± 8.6	23.4 ± 8.7	0.955
Takeuchi II (*n* = 11)	28.0 ± 9.4	28.1 ± 9.5	0.832
Takeuchi III (*n* = 1)	21.9	15.7	*n.a.* *
Ascending osteotomy (*n* = 56)	24.4 ± 1.1	24.2 ± 1.1	0.596
Descending osteotomy (*n* = 62)	25.7 ± 1.1	25.8 ± 1.1	0.891

*n.a., not applicable due to *n* = 1.

**Table 2:** Mean change in tibial torsion angle (TTA) (mean value ± standard deviation).

	Mean change in TTA (°)	*p*‐Value
Overall (*n* = 118)	0.1 ± 3.0	
No HF group (*n* = 66)	0.1 ± 2.8	
HF group (*n* = 52)	0.1 ± 3.3	0.955
Takeuchi I (*n* = 40)	0.0 ± 3.5	0.933
Takeuchi II (*n* = 11)	−0.1 ± 2.0	0.807
Takeuchi III (*n* = 1)	−6.2	n.a.*
Ascending osteotomy (*n* = 56)	0.2 ± 3.5	0.597
Descending osteotomy (*n* = 62)	0.0 ± 2.5	

*Note*: The no HF group served as the reference group for *p*‐value calculation of HF group and subgroup analysis of Takeuchi I, II and III. Comparison between ascending and descending osteotomy behind the tibial tuberosity. *n.a., not applicable due to *n* = 1.

Furthermore, the HF group (9.2 mm ± 2.9 mm) had significantly larger OWD values than no HF group (7.4 mm ± 2.1 mm, *p* < 0.001) and underwent a greater HKA correction (HF group: 8.6° ± 3.7°, no HF group: 8.2° ± 3.8°, *p* = 0.543). The takeuchi classification showed excellent agreement (Cohen's κ: 0.955). The ICC between the two readers showed “almost perfect agreement” for all parameters: tibial torsion (0.95 [95% confidence interval, CI: 0.88–0.98]), preoperative tibial torsion (0.82 [95% CI: 0.74–0.88]), OWD (0.96 [95% CI: 0.94–0.97]), preoperative PTS (0.92 [95% CI: 0.88–0.94]), postoperative PTS (0.90 [95% CI: 0.86–0.93]), preoperative HKA (0.99 [95% CI: 0.96–0.99]), postoperative HKA (0.97 [95% CI: 0.95–0.98]) and Takeuchi classification (0.98 [95% CI: 0.98–0.99]).

## DISCUSSION

The most important finding of this study is that MOW‐HTO performed with the PSI system used in this study showed no significant change in TTA, even in case of a HF. However, the direction of the osteotomy behind the tibial tuberosity, namely ascending or descending, had no influence on postoperative TTA with use of this PSI system.

Reported TTA changes for the conventional technique range from −1.8° ± 3.4° up to −3.0° ± 3.4° [19, 24]. Consequently, several studies have investigated factors contributing to TTA changes after MOW‐HTO and strategies to minimise them. Factors associated with postoperative TTA changes include larger correction angles, medial soft tissue tension, and the osteotomy configuration, with descending osteotomies behind the tibial tuberosity reported to result in smaller rotational changes than ascending osteotomies behind the tibial tuberosity [17, 19, 36]. Among these factors, Jung et al. identified lateral cortical instability, as observed in HF, as one of the main causes of unintended TTA changes [17]. They reported a mean postoperative TTA change of −3.9° ± 4.7° in cases with HF [17]. These findings are supported by biomechanical studies demonstrating progressively reduced construct stability with increasing severity of HF according to the Takeuchi classification [4, 35]. Current literature reports incidences of HF up to 59% [18]. HF should therefore be avoided because it is associated with a reduced construct stiffness, a higher rate of nonunion, loss of correction, implant failure and TTA changes [11, 27, 31, 33]. Several strategies have been proposed to avoid this complication, such as the use of a protective hinge‐wire, optimising the position of the osteotomy hinge, the cautious opening of the osteotomy and limiting the OWD to no more than 11 mm [4, 10, 20, 30, 32]. Another approach to minimise unintended rotational changes after osteotomy is the use of reduction guides, as implemented in PSI. For distal femoral osteotomies, PSI‐assisted correction has been shown to achieve excellent rotational stability, even in case of HF [15]. However, in a cadaveric study on MOW‐HTO, Jörgens et al. reported a postoperative TTA deviation of 5.74° ± 3.24° despite the use of PSI [12]. When comparing these results, the differences in guide design between PSI systems have to be considered, as these may influence rotational stability. In the system used by Jörgens et al., the cutting guide is removed after the osteotomy and a dedicated PSI spacer is positioned in the osteotomy gap without relying on previously placed reference pins.

In contrast to these previous reports, TTA showed no significant systematic change in TTA from pre‐ to postoperative in the present cohort, including in patients with a HF. However, the absence of a statistically significant mean change does not exclude clinically relevant rotational changes in individual patients. This may be explained by the guide design of the PSI system used in this study (Figure 1). Before the osteotomy is performed, the patient‐specific osteotomy guide is rigidly fixed to the tibia using four 4.0 mm Schanz pins, which serve as permanent reference points throughout the procedure. Following completion of the osteotomy, the reduction guide is mounted onto the same reference pins. By referencing the identical pin configuration before and after the osteotomy, the system enables precise control of fragment reduction and may contributes to the consistent preservation of the TTA observed in this study. The PTS change associated with MOW‐HTO as previously described could also have been replicated in the present cohort (mean change ± SD: −1 ± 2.4, *p* < 0.001) [22, 23, 29]. However, in contrast to the previous literature with improved rotational stability in MOW‐HTO with a descending osteotomy behind the tibial tuberosity compared to the ascending technique [36], in this study, no significant change in TTA was observed between these two techniques, likely because the reduction guide itself provides sufficient stability to maintain fragment position, thereby no additional stabilising effect of supplementary screws is observed.

The following limitations should be considered to contextualise the findings of this study. The primary limitation of this study is the absence of a control group treated with the conventional technique, as PSI is the standard approach at the study institution. Therefore, comparisons of this retrospective single‐arm observational study to conventional techniques or other PSI designs rely on previously published literature and should be interpreted with caution. The results of the direction of the osteotomy behind the tibial tuberosity should be interpreted carefully as the groups were not randomised. Another limitation is that different surgeons performed the procedures, although the senior surgeons (S.F. and L.V.) performed or supervised most of the osteotomies.

## CONCLUSION

No significant change in TTA was observed in MOW‐HTO performed with a PSI system consisting of an osteotomy guide followed by a reduction guide. TTA also remained stable in cases with a hinge fracture.

## AUTHOR CONTRIBUTIONS

All authors have made substantial contributions to all of the following: (1) the conception and design of the study, or acquisition of data, or analysis and interpretation of data (2), drafting the article or revising it critically for important intellectual content (3), final approval of the version to be submitted.

## FUNDING

The authors have no funding to report.

## CONFLICT OF INTEREST STATEMENT

The authors declare no conflicts of interest.

## ETHICS STATEMENT

The local ethical committee approved this retrospective study (Zurich Cantonal Ethics Commission, BASEC‐Nr. 2023‐00389). Informed consent was obtained from all study participants.

## Data Availability

The data that support the findings of this study are available on request from the corresponding author. The data are not publicly available due to privacy or ethical restrictions.
